# Multiple Linear-Combination Security Network Coding

**DOI:** 10.3390/e25081135

**Published:** 2023-07-28

**Authors:** Yang Bai, Xuan Guang, Raymond W. Yeung

**Affiliations:** 1 School of Mathematical Sciences and LPMC, Nankai University, Tianjin 300071, China; 2 Institute of Network Coding and the Department of Information Engineering, The Chinese University of Hong Kong, Hong Kong SAR, China

**Keywords:** information-theoretical security, linear-combination security, network coding, secure network coding, security capacity, code construction, asymptotic behavior

## Abstract

In this paper, we put forward the model of multiple linear-combination security multicast network coding, where the wiretapper desires to obtain some information about a predefined set of multiple linear combinations of the source symbols by eavesdropping any one (but not more than one) channel subset up to a certain size *r*, referred to as the *security level*. For this model, the security capacity is defined as the maximum average number of source symbols that can be securely multicast to all sink nodes for one use of the network under the linear-combination security constraint. For any security level and any linear-combination security constraint, we fully characterize the security capacity in terms of the ratio of the rank of the linear-combination security constraint to the number of source symbols. Also, we develop a general construction of linear security network codes. Finally, we investigate the asymptotic behavior of the security capacity for a sequence of linear-combination security models and discuss the asymptotic optimality of our code construction.

## 1. Introduction

In 2000, Ahlswede et al. [1] proposed the general concept of network coding. In particular, they investigated the single-source multicast network coding problem, where the source symbols generated by a single source node are required to multicast to multiple sink nodes through a noiseless network while the nodes in the network are allowed to process the received information. It was proven in [1] that if coding is applied at the intermediate nodes (rather than routing only), the source node can multicast source symbols to all the sink nodes at the theoretically maximum rate, i.e., the smallest minimum cut capacity separating a sink node from the source node, as the alphabet size of both the information source and the channel transmission symbol tends toward infinity. In 2003, Li et al. [2] proved that linear network coding over a finite alphabet is sufficient for optimal multicast by means of a vector space approach. Independently, Koetter and Médard [3] developed an algebraic characterization of linear network coding by means of a matrix approach. Jaggi et al. [4] further presented a deterministic polynomial-time algorithm for constructing a linear network code. For comprehensive discussions of network coding, we refer the reader to [5,6,7,8,9,10].

In the paradigm of network coding, information-theoretic security in the presence of a wiretapper is naturally considered (cf. [11,12,13,14,15,16,17,18,19,20,21,22,23,24,25,26,27,28]), called the *secure network coding* problem. In the model of secure network coding over a wiretap network, (i) the source node multicasts the source symbols to all the sink nodes, which, as legal users, are required to correctly decode the source symbols; and (ii) the wiretapper, who can access any one but not more than one wiretap set of communication channels, is not allowed to obtain any information about the source symbols. The classical information-theoretically secure models, e.g., Shannon’s cipher system [29], secret sharing [30,31] and the wiretap channel II [32], can be regarded as special cases of the secure network coding model. In particular, a wiretap network is called an *r*-*wiretap network* if the wiretapper can fully access an arbitrary subset of, at most, *r* edges, where the non-negative integer *r* is called the *security level*.

In the model of secure network coding, to guarantee the required information-theoretic security, it is necessary to randomize the source symbols to combat the wiretapper. Cai and Yeung [11] presented a code construction for the *r*-wiretap network. El Rouayheb et al. [12] further showed that the Cai–Yeung code construction can be viewed as a network generalization of the code construction for wiretap channel II in [32]. Motivated by El Rouayheb et al., Silva and Kschischang [13] proposed a universal design of security network codes based on rank-metric codes. For the construction of security network codes in [11,12,13]. However, the existing upper bounds on the minimum required alphabet size may be too large for implementation for certain applications in terms of computational complexity and storage requirement. Feldman et al. [33] showed that for a given security level, the alphabet size can be reduced by sacrificing a small fraction of the information rate. However, if the information rate is not sacrificed, whether it is possible to reduce the required alphabet size is considered an open problem [12,17]. Recently, Guang and Yeung [18] developed a systematic graph-theoretic approach to improve the upper bound on the minimum required alphabet size for the existence of secure network codes, achieving an improvement of general significance. Subsequently, in order to tackle the problem of secure network coding when the information rate and the secure level may vary over time, Guang et al. [19] put forward local-encoding-preserving secure network coding, where a family of secure linear network codes is called local-encoding-preserving if all the codes in this family use a common local encoding operation at each intermediate node in the network. They also constructed a family of local-encoding-preserving secure linear network codes applicable for all possible pairs of rate and security level. We note that the variable-rate linear network coding problem without security consideration was previously investigated by Fong and Yeung [34].

In this paper, we put forward the model of multiple linear-combination security network coding, where multiple linear combinations (containing single linear combination as a special case) of the source symbols are required to be protected from the wiretapper. More precisely, in this model over an *r*-wiretap network, (i) the single source node generates source symbols over a finite field *F*, and all the source symbols are required to be correctly decoded at all the sink nodes; and (ii) for a predefined set of linear combinations of the source symbols, the wiretapper, who can fully access any channel subset of a size not larger than *r*, is not allowed to obtain any information about these linear combinations. For the above security model with security level *r*, the (linear-combination) security capacity is defined as the maximum average number of source symbols that can be securely multicast to all the sink nodes for one use of the network under the above linear-combination security constraint. A model related to the current work is that considered by Bhattad and Narayanan [23], which contains weakly secure network coding as a special case. The relation between the current work and that of Bhattad and Narayanan [23] is discussed in Appendix A.

In this paper, we investigate the security capacity and the code construction for this model and analyze the asymptotic behavior of the security capacity and code construction for a sequence of linear-combination security models. The main contributions and organization of this paper are as follows:

- In Section 2, we formally present the model of linear-combination security network coding and the preliminaries, including the necessary notation and definitions.
- In Section 3, we characterize the security capacity by considering different cases of the security level *r*. We first prove that $C_{\min}-1$ is the maximum security level such that the source symbols can be securely multicast to all sink nodes with a positive rate, where $C_{\min}$ is the smallest minimum cut capacity separating a sink node from the source node. Therefore, the security capacity is zero for $r\geq C_{\min}$. For any nontrivial security level $1\leq r\leq C_{\min}-1$, we prove upper bounds on the security capacity in terms of the ratio τ of the rank of the linear-combination security constraint to the number of source symbols.
  We further develop a systematic construction of linear security network codes, which is applicable to an arbitrary linear-combination security model. Based on the obtained upper bounds and the developed code construction, we fully characterize the security capacity for any possible pair of the number of the source symbols and the linear-combination security constraint. We also determine the threshold value $\tau_{0}$ such that there is no penalty on the security capacity compared with the capacity without any security consideration when the ratio τ is not larger than $\tau_{0}$.
- In Section 4, we fully characterize the asymptotic behavior of the security capacity for a sequence of linear-combination security models and prove that our code construction is asymptotically optimal.
- We conclude in Section 5 with a summary of our results.

## 2. Preliminaries

Consider a communication network whose communication channels are point-to-point. The network is represented by a directed acyclic graph G=(V,E), where V and E are finite sets of nodes and edges, respectively. Here, an edge in the graph G corresponds to a point-to-point channel in the network. In the graph G, multiple edges between two nodes are allowed. We assume that an element in a finite field *F* can be reliably transmitted on each edge for each use. We use tail(e) and head(e) to denote the *tail* node and the *head* node of an edge *e*, respectively. For a node v∈V, we let In(v)={e∈E:head(e)=v} and Out(v)={e∈E:tail(e)=v}, i.e., In(v) and Out(v) are the set of input edges and the set of output edges, respectively. Furthermore, a sequence of edges $\left(e_{1},e_{2},\cdots ,e_{m}\right)$ is called a (directed) *path* from the node $\mathrm{tail}(e_{1})$ to the node $\mathrm{head}(e_{m})$ if $\mathrm{tail}\left(e_{i}\right)=\mathrm{head}\left(e_{i-1}\right)$ for i=2,3,⋯,m. For two nodes *u* and *v* with u≠v, an edge subset C⊆E is called a *cut* separating *v* from *u* if no path exists from *u* to *v* upon removing the edges in *C*. The *capacity* of a cut separating *v* from *u* is defined as the size of this cut. A cut *C* separating *v* from *u* is called a *minimum cut* separating *v* from *u* if there does not exist a cut $C^{\prime }$ separating *v* from *u* such that $|C^{\prime }\left|<|C\right|$. The capacity of a minimum cut separating *v* from *u* is called the *minimum cut capacity* separating *v* from *u*, as denoted by mincut(u,v). There is a single *source node* s∈V and a set of *sink nodes* T⊆V∖{s} on the graph G. Without loss of generality, we assume that the source node *s* has no input edges and that every sink node t∈T has no output edges, i.e., In(s)=Out(t)=∅,∀t∈T. The graph G, together with *s* and *T*, forms a *network* N denoted by N=(G,s,T).

The source node *s* generates *Lsource symbols* $B_{1},B_{2},\cdots ,B_{L}$ that are independent and identically distributed (i.i.d.) random variables with a uniform distribution on the finite field *F*. All the source symbols are required to be multicast to every sink node *t* in *T* by using the network N multiple times, i.e., transmitting multiple elements in *F* on each edge by using the edge multiple times. There is a wiretapper who can eavesdrop any edge subset of a size up to the security level *r*, while, for a positive integer $m_{L}$, the $m_{L}$ linear combinations of the source symbols

(1) $$\sum_{i=1}^{L}a_{i,j}\cdot B_{i},\;\;j=1,2,\cdots ,m_{L}$$

over the finite field *F* are required to be protected from the wiretapper, where $a_{i,j},\;1\leq i\leq L,1\leq j\leq m_{L}$ are constants in *F*; that is, the wiretapper is not allowed to obtain any information about the multiple linear combinations of the source symbols given in (1). Furthermore, we let $B=(B_{1},B_{2},\cdots ,B_{L})$, and

$$M_{L}={\left[a_{i,j}\right]}_{1\leq i\leq L,\;1\leq j\leq m_{L}},$$

is an $L\times m_{L}$ matrix. Then, the $m_{L}$ linear combinations in (1) can be written as $B\;\cdot \;M_{L}$. Without loss of generality, we assume that $m_{L}\leq L$ and that the matrix $M_{L}$ has full column rank, i.e.,

$$\mathrm{Rank}\left(M_{L}\right)=m_{L}.$$

In this model, the security level *r* is known by the source node and sink nodes, but which edge subset is eavesdropped by the wiretapper is unknown. It suffices to consider only the wiretap sets of a size exactly equal to *r*. Then, we let

$$W_{r}≜\left\{W\subseteq E:\;|W|=r\right\},$$

where each edge subset $W\in W_{r}$ is called a *wiretap set*. We use $\left\{\left(L,M_{L}\right),r\right\}$ to denote the above linear-combination security model.

Next, we define a *(linear-combination) security network code* for the security model $\left\{\left(L,M_{L}\right),r\right\}$. In order to combat the wiretapper, we may need randomness to randomize the source symbols. However, as we show, it is not always necessary to randomize the source symbols. As part of the code to be defined, we assume that the *key* K is a random variable uniformly distributed over a finite set K, which is available only at the source node *s*. The key K and the source symbols $B_{i},\;i=1,2,\cdots ,L$ are assumed to be mutually independent. A $\left(L,M_{L}\right)$ security network code is defined as follows. First, we let $b_{i}\in F$ and k∈K be arbitrary outputs of the source symbol $B_{i}$ and the key K, respectively, i=1,2,⋯,L. We further let $b=(b_{1},b_{2},\cdots ,b_{L})$, which is the output of the vector of source symbols $B=(B_{1},B_{2},\cdots ,B_{L})$. A $\left(L,M_{L}\right)$ security network code $\hat{C}$ consists of:

- A *local encoding function* ${\hat{\theta }}_{e}$ for each edge e∈E, where
  (2) θ^e:FL×K→Im(θ^e),iftail(e)=s;∏d∈In(tail(e))Im(θ^d)→Im(θ^e),otherwise;
  with Im(θ^e) denoting the image set of ${\hat{\theta }}_{e}$;
- A decoding function for each sink node t∈T:
  φ^t:∏e∈In(t)Im(θ^e)→FL
  to decode the source symbols $b_{1},b_{2},\cdots ,b_{L}$ at *t*.

Furthermore, we use ye∈Im(θ^e) to denote the message transmitted on each edge e∈E by using the code $\hat{C}$ under b and k. With the encoding mechanism described in (2), we readily see that $y_{e}$ is a function of b and k, as denoted by ${\hat{h}}_{e}\left(b\;k\right)$ (i.e., $y_{e}={\hat{h}}_{e}\left(b\;k\right)$), where ${\hat{h}}_{e}$ can be obtained by recursively applying the local encoding functions ${\hat{\theta }}_{e},e\in E$ according to any ancestral order of the edges in E. More precisely, for each e∈E, we have

$${\hat{h}}_{e}\left(b\;k\right)=\left\{\begin{array}{cc} {\hat{\theta }}_{e}\left(b\;k\right), & \mathrm{if}\mathrm{tail}(e)=s;\\ {\hat{\theta }}_{e}\left({\hat{h}}_{\mathrm{In}(u)}\left(b\;k\right)\right), & \mathrm{otherwise}; \end{array}\right.$$

where u=tail(e) and ${\hat{h}}_{E}\left(b\;k\right)≜\left({\hat{h}}_{e}\left(b\;k\right):e\in E\right)$ for an edge subset E⊆E so that ${\hat{h}}_{\mathrm{In}(u)}\left(b\;k\right)=\left({\hat{h}}_{e}\left(b\;k\right):e\in \mathrm{In}\left(u\right)\right)$. We call ${\hat{h}}_{e}$ the *global encoding function* of the edge *e* for the code $\hat{C}$.

For the security model $\left\{\left(L,M_{L}\right),r\right\}$, a $\left(L,M_{L}\right)$ security network code $\hat{C}=\left\{{\hat{\theta }}_{e}:e\in E;\;{\hat{\varphi }}_{t}:t\in T\right\}$ is *admissible* if the following *decoding* and *security conditions* are satisfied:

- ***Decoding condition***: All the source symbols are correctly decoded for each sink node t∈T, i.e., for each t∈T,
  (3) $${\hat{\varphi }}_{t}\left({\hat{h}}_{\mathrm{In}(t)}\left(b\;k\right)\right)=b,\;\forall \;b\in F^{L}\;\;\mathrm{and}\;\;\forall \;k\in K;$$
- ***Security condition***: for each wiretap set, $W\in W_{r}$,
  (4) $$I\left(Y_{W};\;B\;\cdot \;M_{L}\right)=0,$$
  where $Y_{W}≜\left(Y_{e}:e\in W\right)$, and $Y_{e}≜{\hat{h}}_{e}\left(B,K\right)$ is the random variable transmitted on the edge *e*.

For an admissible $\left(L,M_{L}\right)$ security network code $\hat{C}=\left\{{\hat{\theta }}_{e}:e\in E;\;{\hat{\varphi }}_{t}:t\in T\right\}$, we let

ne=log|F||Im(θ^e)|

for each edge *e* in E, which is regarded as the number of times the edge *e* is used for transmission when applying the code $\hat{C}$. We further let $n\left(\hat{C}\right)≜\max_{e\in E}n_{e}$. Then, the *rate* of $\hat{C}$ is defined by

(5) $$R\left(\hat{C}\right)=\frac{L}{n(\hat{C})},$$

which is the average number of source symbols that can be securely multicast to all the sink nodes for one use of the network using the code $\hat{C}$.

Furthermore, the *security capacity* for this model $\left\{\left(L,M_{L}\right),r\right\}$ is defined as the maximum rate of all admissible $\left(L,M_{L}\right)$ security network codes, i.e.,

$$C=\max\left\{R\left(\hat{C}\right):\hat{C}\;\mathrm{is}\;\mathrm{an}\;\mathrm{admissible}\;\left(L,M_{L}\right)\;\sec\mathrm{urity}\;\mathrm{network}\;\mathrm{code}\;\mathrm{for}\;\left\{\left(L,M_{L}\right),r\right\}\right\}.$$

According to the definition of the rate in (5), characterizing the security capacity C is equivalent to determining the minimum $n(\hat{C})$ over all the admissible $\left(L,M_{L}\right)$ security network codes, i.e.,

$$n^{*}≜\min\left\{n\left(\hat{C}\right):\hat{C}\;\mathrm{is}\;\mathrm{an}\;\mathrm{admissible}\;\left(L,M_{L}\right)\;\mathrm{security}\;\mathrm{network}\;\mathrm{code}\;\mathrm{for}\;\left\{\left(L,M_{L}\right),r\right\}\right\}.$$

For instance, a special case of the linear-combination security model $\left\{\left(L,M_{L}\right),r\right\}$ is algebraic-sum security network coding, as elaborated below. In this model, the source node *s* generates *L* source symbols $B_{1},B_{2},\cdots ,B_{L}$, which are required to be multicast to every sink node t∈T, and the wiretapper, who can eavesdrop any edge subset of size *r*, is not allowed to obtain any information about the *m* algebraic sums of the source symbols:

(6) ∑i∈[L]:i≡j(modm)Bi,j=1,2,⋯,m,

where 1≤m≤L, and [L]≜{1,2,⋯,L}. For this algebraic-sum security model, when m=1, we adopt the convention that i≡1(mod1) for all i=1,2,⋯,L. Then, Equation (6) becomes $\sum_{i=1}^{L}B_{i}$, i.e., the algebraic sum $\sum_{i=1}^{L}B_{i}$ of all the *L* source symbols is required to be protected from the wiretapper. When m=L, we have $i≢i^{\prime }\left(\mathrm{mod}\;m\right),\;\forall \;i,i^{\prime }\in \left[L\right]$, where $i\neq i^{\prime }$; thus, all the source symbols $B_{1},B_{2},\cdots ,B_{L}$ are required to be protected from the wiretapper. This is the standard model of secure network coding, which has been widely studied in the literature, e.g., [11,12,13,14,15,16,17,18,19,20,21,22,23,24].

An example scenario for the application of the linear-combination security model is as follows. A predefined set of linear combinations of the source symbols is required to be protected from the wiretapper, while other linear combinations are unprotected. The source node *s* generates *L* source symbols $B_{1},B_{2},\cdots ,B_{L}$ in the finite field *F*, which are required to be multicast to every sink node t∈T. The $L\times m_{L}$ matrix $M_{L}$ is regarded as an *F*-valued parity-check matrix. We denote the solution space of the system of linear equations $\vec{b}\cdot M_{L}=\vec{0}$ over *F* as $V(\vec{0})$, i.e.,

$$V\left(\vec{0}\right)=\left\{\vec{b}\in F^{L}:\;\vec{b}\cdot M_{L}=\vec{0}\right\},$$

where $\vec{0}$ is the zero row $m_{L}$-vector. According to the value of $\vec{b}\cdot M_{L}$, for every output $\vec{b}\in F^{L}$ of $B=(B_{1},B_{2},\cdots ,B_{L})$, the vector space $F^{L}$ can be partitioned into ${\left|F\right|}^{m_{L}}$ cosets of the solution space given by $\left\{V\left(\vec{a}\right):\;\vec{a}\in F^{m_{L}}\right\}$, where $V\left(\vec{a}\right)≜\left\{\vec{b}\in F^{L}:\;\vec{b}\cdot M_{L}=\vec{a}\right\}.$ In this scenario, we desire to protect the information as to which coset $V(\vec{a})$ the vector $\vec{b}$ lies in, which may contain some useful information for the wiretapper. In other words, the information about the specified linear combinations $B\cdot M_{L}$ needs to be protected from the wiretapper, while other linear combinations are unprotected.

## 3. Characterization of the Capacity ofthe Security Model $\left\{\left(L,M_{L}\right),r\right\}$

### 3.1. Upper Bounds on the Security Capacity

Consider a linear-combination security model $\left\{\left(L,M_{L}\right),r\right\}$. We first consider the trivial case of $r\geq C_{\min}$, where $C_{\min}≜\min_{t\in T}\mathrm{mincut}\left(s,t\right)$. In this case, for a sink node t∈T such that $\mathrm{mincut}\left(s,t\right)=C_{\min}$, the wiretapper is able to decode the source symbols, provided that the sink node *t* correctly decodes them. This shows that the security capacity is C=0 for $r\geq C_{\min}$, which implies that $C_{\min}-1$ is an upper bound on the maximum security level for which the source symbols can be multicast with a positive rate. For another trivial case r=0, the security model $\left\{\left(L,M_{L}\right),0\right\}$ becomes a single-source multicast network coding problem without any security consideration. Given the fact that the maximum rate at which the source symbols can be correctly multicast to all the sink nodes is $C_{\min}$ (cf. [1,6]), we thus obtain that

$$n^{*}=\left\lceil \frac{L}{C_{\min}}\right\rceil ,$$

or, equivalently,

$$C=\frac{L}{n^{*}}=\frac{L}{\left\lceil L/C_{\min}\right\rceil }.$$

Next, we consider $0<r<C_{\min}$. We readily see that an admissible $\left(L,M_{L}\right)$ security network code $\hat{C}$ is also a network code such that all the *L* source symbols can be correctly decoded at each t∈T. This immediately implies that $n^{*}$ can be lower-bounded by $\left\lceil L/C_{\min}\right\rceil$ for any security level $0<r<C_{\min}$, i.e.,

(7) $$n^{*}\geq \left\lceil \frac{L}{C_{\min}}\right\rceil .$$

Furthermore, we present the following lemma, which asserts a non-trivial lower bound on $n^{*}$.

**Lemma 1.**  *Consider a linear-combination security model $\left\{\left(L,M_{L}\right),r\right\}$ with a security level of $0<r<C_{\min}$, where $\mathrm{Rank}\left(M_{L}\right)=m_{L}$. Let $\tau =m_{L}/L$. Then,*

(8) $$n^{*}\geq \left\lceil \frac{\tau L}{C_{\min}-r}\right\rceil .$$

**Proof.**  First, we claim that

(9) $$H(B\cdot M_{L})=\tau L\cdot \log|F|,$$

where $\tau L=m_{L}$. To see this, we consider an arbitrary row vector $\vec{x}\in F^{\tau L}$ and obtain

(10) $$\begin{array}{cc} \mathrm{Pr}\left(B\;\cdot \;M_{L}=\vec{x}\right) & =\sum_{b\in F^{L}:\;b\cdot M_{L}=\vec{x}}\mathrm{Pr}\left(B=b\right)\\ \; & =\#\left\{b\in F^{L}:b\;\cdot \;M_{L}=\vec{x}\right\}\cdot \frac{1}{{\left|F\right|}^{L}}=\frac{1}{{\left|F\right|}^{\tau L}}, \end{array}$$

where the equality $\mathrm{Pr}\left(B=b\right)=\frac{1}{{\left|F\right|}^{L}}$ holds because the source symbols $B_{i},1\leq i\leq L$ are i.i.d. with the uniform distribution on *F*.

We now consider an arbitrary admissible $\left(L,M_{L}\right)$ security network code: $\hat{C}=\left\{{\hat{\theta }}_{e}:e\in E;\;{\hat{\varphi }}_{t}:t\in T\right\}$. For an edge subset *C* that separates a sink node t∈T from the source node *s*, it follows from the decoding condition (3) that $H(B|Y_{C})=0$. This immediately implies that

(11) $$\begin{array}{c} H\left(B\cdot M_{L}|Y_{C}\right)=0. \end{array}$$

Furthermore, for any wiretap set $W\in W_{r}$ with W⊆C, it follows from the security condition (4) that

(12) $$\begin{array}{c} H\left(B\cdot M_{L}\right)=H\left(B\cdot M_{L}|Y_{W}\right). \end{array}$$

Combining (11) and (12), we obtain

$$\begin{array}{cc} \;\;H\left(B\cdot M_{L}\right) & =H\left(B\cdot M_{L}|Y_{W}\right)-H\left(B\cdot M_{L}|Y_{C}\right)\\ \; & =I\left(B\cdot M_{L};\;Y_{C\setminus W}|Y_{W}\right)\\ \; & \leq H\left(Y_{C\setminus W}|Y_{W}\right)\\ \; & \leq H\left(Y_{C\setminus W}\right)\\ \; & \leq \sum_{e\in C\setminus W}H\left(Y_{e}\right) \end{array}$$

(13)  ≤∑e∈C∖Wlog|Im(θ^e)|

(14) $$\begin{array}{cc} \; & \leq \sum_{e\in C\setminus W}n_{e}\cdot \log\left|F\right| \end{array}$$

(15) $$\begin{array}{cc} \; & \;\;\leq n(\hat{C})\cdot |C\setminus W|\cdot \log|F|, \end{array}$$

where the inequality (13) holds because $Y_{e}$ takes values in Im(θ^e), and the inequality (14) follows from

ne=log|F||Im(θ^e)|≥log|F||Im(θ^e)|,

and the inequality (15) follows from $n\left(\hat{C}\right)=\max_{e\in E}n_{e}$.

Combining (9) and (15), we obtain

$$\begin{array}{c} n\left(\hat{C}\right)\geq \frac{H\left(B\cdot M_{L}\right)}{\left|C\setminus W|\cdot \log|F\right|}=\frac{\tau L}{\left|C\setminus W\right|}. \end{array}$$

Note that the above inequality is true for each sink node t∈T and all the pairs (C,W) of the cut *C* separating *t* from *s* and the wiretap set $W\in W_{r}$ such that W⊆C. We thus obtain

n(C^)≥maxt∈Tmax(W,C)∈Wr×Λt:W⊆CτL|C∖W|,

where $\Lambda_{t}≜\left\{C\subseteq E:C\;\mathrm{is}\;a\;\mathrm{cut}\;\mathrm{separating}\;t\;\mathrm{from}\;s\right\}$. For each t∈T, we have

$$\begin{array}{c} \left|C\setminus W\right|\geq C_{\min}-r,\;\forall \;\left(W,C\right)\in W_{r}\times \Lambda_{t}\;\mathrm{with}\;W\subseteq C. \end{array}$$

According to the definition of $C_{\min}$, this lower bound is achievable for some t∈T and $\left(W,C\right)\in W_{r}\times \Lambda_{t}$ such that W⊆C. It then follows that

$$n\left(\hat{C}\right)\geq \frac{\tau L}{C_{\min}-r}.$$

Furthermore, since $n(\hat{C})$ is an integer, we have

(16) $$n\left(\hat{C}\right)\geq \left\lceil \frac{\tau L}{C_{\min}-r}\right\rceil .$$

In addition, because the above lower bound (16) on $n(\hat{C})$ is valid for any admissible $\left(L,M_{L}\right)$ security network code $\hat{C}$, we obtain

$$n^{*}\geq \left\lceil \frac{\tau L}{C_{\min}-r}\right\rceil .$$

The lemma is thus proven. □

The lower bounds in (7) and (8) on $n^{*}$ apply to all $0<r<C_{\min}$. For a specific value of τ, one of them can be tighter than the other. By comparing these bounds, we can readily obtain the upper bounds on the security capacity C as stated in the following theorem.

**Theorem 1.**  *Consider a linear-combination security model $\left\{\left(L,M_{L}\right),r\right\}$ with a security level of $0<r<C_{\min}$, where $\mathrm{Rank}\left(M_{L}\right)=m_{L}$. Let*

$$\tau =\frac{m_{L}}{L}\;\;\mathrm{and}\;\;\tau_{0}=\frac{C_{\min}-r}{C_{\min}}.$$

- *If $0\leq \tau \leq \tau_{0}$, then*
$$C\leq \frac{L}{\left\lceil L/C_{\min}\right\rceil }.$$
- *If $\tau_{0}<\tau \leq 1$, then*
$$C\leq \frac{L}{\left\lceil \tau L/(C_{\min}-r)\right\rceil }.$$

**Proof.**  By comparing the lower bounds ((7) and (8)) on $n^{*}$, we immediately obtain

- if $0\leq \tau \leq \tau_{0}$, then
  (17) $$n^{*}\geq \left\lceil \frac{L}{C_{\min}}\right\rceil \geq \left\lceil \frac{\tau L}{C_{\min}-r}\right\rceil$$
  implying that
  $$C\leq \frac{L}{\left\lceil L/C_{\min}\right\rceil };$$
- If $\tau_{0}<\tau \leq 1$, we have
  (18) $$n^{*}\geq \left\lceil \frac{\tau L}{C_{\min}-r}\right\rceil \geq \left\lceil \frac{L}{C_{\min}}\right\rceil$$
  implying that
  $$C\leq \frac{L}{\left\lceil \tau L/C_{\min}\right\rceil }.$$

We have thus proven the theorem. □

### 3.2. Characterization of the Security Capacity

Next, we present a code construction for the security model $\left\{\left(L,M_{L}\right),r\right\}$ with $0<r<C_{\min}$, which shows that the upper bounds in Theorem 1 for both cases of τ are tight. We thus obtain a full characterization of the security capacity for the security model $\left\{\left(L,M_{L}\right),r\right\}$, as stated in the following theorem.

**Theorem 2.**  *Consider a linear-combination security model $\left\{\left(L,M_{L}\right),r\right\}$ over a finite field F, where $0<r<C_{\min}$ and |F|>max|T|,|E|r. Let*

$$\tau =\frac{m_{L}}{L}\;\;\mathrm{and}\;\;\tau_{0}=\frac{C_{\min}-r}{C_{\min}}.$$

- *If $0\leq \tau \leq \tau_{0}$, then*
(19) $$C=\frac{L}{\left\lceil L/C_{\min}\right\rceil }.$$
- *If $\tau_{0}<\tau \leq 1$, then*
(20) $$C=\frac{L}{\left\lceil \tau L/(C_{\min}-r)\right\rceil }.$$

This theorem reveals the somewhat surprising fact that for the case of $0\leq \tau \leq \tau_{0}$, there is no penalty on the security capacity compared with the capacity without any security consideration. In Section 4, we further investigate the asymptotic behavior of the security capacity for a sequence of the security models as *L* tends toward infinity. We not only characterize the asymptotic behavior of the security capacity but also show the asymptotic optimality of our construction.

We first define a linear security network code for the security model $\left\{\left(L,M_{L}\right),r\right\}$. Briefly, a $\left(L,M_{L}\right)$ security network code $\hat{C}$ is *linear* if the local encoding functions for all the edges are linear. Specifically, we recall that $b=\left(b_{1},b_{2},\cdots ,b_{L}\right)\in F^{L}$ is an arbitrary output of the vector of source symbols $B=(B_{1},B_{2},\cdots ,B_{L})$. Let $K=F^{z}$, where the non-negative integer *z* is specified later. Then, the key K is a random row vector uniformly distributed on $F^{z}$. We further let $k\in F^{z}$ be an arbitrary output of K. Consequently, for a $\left(L,M_{L}\right)$ linear security network code $\hat{C}$, all the global encoding functions ${\hat{h}}_{e},\;e\in E$ are linear functions of b and k. Therefore, there exists an *F*-valued (L+z)×n matrix $H_{e}=\left[{\vec{h}}_{e}^{\left(1\right)}\;{\vec{h}}_{e}^{\left(2\right)}\;\cdots \;{\vec{h}}_{e}^{\left(n\right)}\right]$ for each e∈E such that

$$\begin{array}{c} {\hat{h}}_{e}\left(b\;k\right)=\left(b\;k\right)\cdot H_{e}, \end{array}$$

where $n≜n(\hat{C})$, and $H_{e}$ is called the *global encoding matrix* of the edge *e* for the code $\hat{C}$. In particular, if $n(\hat{C})=1$, then the code $\hat{C}$ is called a $\left(L,M_{L}\right)$ *scalar-linear* security network code.

In the following, for the nontrivial case of a security model $\left\{\left(L,M_{L}\right),r\right\}$ with a security level of $0<r<C_{\min}$, we develop a construction of admissible $\left(L,M_{L}\right)$ linear security network codes that can be applied to any pair $\left(L,M_{L}\right)$. This code construction shows that the upper bounds in Theorem 1 for both cases of τ are tight, which we state in the following theorem.

**Theorem 3.**  *Consider a linear-combination security model $\left\{\left(L,M_{L}\right),r\right\}$ over a finite field F, where $\mathrm{Rank}\left(M_{L}\right)=m_{L}$, $0<r<C_{\min}$ and |F|>max|T|,|E|r. Let*

$$\tau =\frac{m_{L}}{L}\;\;\mathrm{and}\;\;\tau_{0}=\frac{C_{\min}-r}{C_{\min}}.$$

*Then, there exists an admissible $\left(L,M_{L}\right)$ linear security network code $\hat{C}$ such that*

- *If $0\leq \tau \leq \tau_{0}$, then*
(21) $$n\left(\hat{C}\right)=\left\lceil \frac{L}{C_{\min}}\right\rceil ;$$
- *if $\tau_{0}<\tau \leq 1$, then*
(22) $$n\left(\hat{C}\right)=\left\lceil \frac{\tau L}{C_{\min}-r}\right\rceil .$$

### 3.3. Proof of Theorem 3

In this subsection, we provide the proof of Theorem 3, which includes three parts: code construction, verification of the decoding condition and verification of the security condition.

▸ **Code construction**:

We consider a linear-combination security model $\left\{\left(L,M_{L}\right),r\right\}$ over a finite field *F*, where $0<r<C_{\min}$ and |F|>max|T|,|E|r. In the following, we construct an admissible $\left(L,M_{L}\right)$ linear security network code such that the *L* source symbols can be securely multicast to all the sink nodes by transmitting *n* symbols on each edge, i.e., using the network *n* times, where

(23) $$n=\left\{\begin{array}{cc} \left\lceil \frac{L}{C_{\min}}\right\rceil , & \mathrm{if}0\leq \tau \leq \tau_{0},\\ \left\lceil \frac{\tau L}{C_{\min}-r}\right\rceil . & \mathrm{if}\tau_{0}<\tau \leq 1, \end{array}\right.$$

(cf. (21) and (22)). For any 0≤τ≤1, we let

(24) $$z=\left\{\begin{array}{cc} 0, & \mathrm{if}L\geq nr+\tau L,\\ nr+\tau L-L, & \mathrm{if}L<nr+\tau L, \end{array}\right.$$

i.e.,

$$K=\left\{\begin{array}{cc} \emptyset , & \mathrm{if}L\geq nr+\tau L,\\ F^{nr+\tau L-L}, & \mathrm{if}L<nr+\tau L. \end{array}\right.$$

According to (24), when L≥nr+τL, it is unnecessary to randomize the source symbols to guarantee linear-combination security. Furthermore, for any pair (L,z) satisfying (24), we observe that

(25) $$nr+\tau L\leq L+z\leq nC_{\min}.$$

The first inequality in (25) is straightforward. To prove the second inequality, we consider two cases below.

**Case 1:**  L≥nr+τL.

According to (24) we have z=0, and thus:

(26) L+z=L.

Furthermore, it follows from (23) that for $0\leq \tau \leq \tau_{0}$,

$$n=\left\lceil \frac{L}{C_{\min}}\right\rceil \geq \frac{L}{C_{\min}};$$

and for $\tau_{0}<\tau \leq 1$,

$$n=\left\lceil \frac{\tau L}{C_{\min}-r}\right\rceil \geq \left\lceil \frac{L}{C_{\min}}\right\rceil \geq \frac{L}{C_{\min}}$$

(cf. (18) for the first inequality in the above equation). Together with (26), we immediately prove that $L+z=L\leq nC_{\min}$ for this case.

**Case 2:**  L<nr+τL.

According to (24), we have

(27) L+z=nr+τL.

Furthermore, it follows from (23) that for $0\leq \tau \leq \tau_{0}$,

$$n=\left\lceil \frac{L}{C_{\min}}\right\rceil \geq \left\lceil \frac{\tau L}{C_{\min}-r}\right\rceil \geq \frac{\tau L}{C_{\min}-r}$$

(cf. (17) for the first inequality in the above equation), and for $\tau_{0}<\tau \leq 1$,

$$n=\left\lceil \frac{\tau L}{C_{\min}-r}\right\rceil \geq \frac{\tau L}{C_{\min}-r}.$$

Together with (27), we immediately obtain that $L+z=nr+\tau L\leq nC_{\min}$ for this case. Combining the two cases, we have proven the second inequality in (25).

According to (25), we have $L+z\leq nC_{\min}$. This implies that the L+z symbols in *F* generated by the source node *s*, which contain the *L* source symbols and the key of *z* symbols, can be multicast to all the sink nodes in *T* by using the network *n* times. To elaborate this, we first claim that

(28) $$L+z>\left(n-1\right)C_{\min}.$$

- When $0\leq \tau \leq \tau_{0}$, it follows from (23) that
  $$\begin{array}{cc} \left(n-1\right)C_{\min} & =\left(\left\lceil \frac{L}{C_{\min}}\right\rceil -1\right)\cdot C_{\min}\\ \; & <\frac{L}{C_{\min}}\cdot C_{\min}=L\leq L+z. \end{array}$$
- When $\tau_{0}<\tau \leq 1$, according to (23), we obtain
  $$\begin{array}{cc} \left(n-1\right)C_{\min} & =\left(\left\lceil \frac{\tau L}{C_{\min}-r}\right\rceil -1\right)\cdot C_{\min}\\ \; & <\frac{\tau L}{C_{\min}-r}\cdot C_{\min}\\ \; & =\tau L+\frac{\tau L}{C_{\min}-r}\cdot r\\ \; & \leq \tau L+nr\leq L+z, \end{array}$$
  where the last two inequalities follow from (23) and (25), respectively.

Thus, we have proven (28).

Now, we let $b_{1}^{\prime },b_{2}^{\prime },\cdots ,b_{L+z}^{\prime }$ be the L+z source symbols, and divide them into *n* groups $b_{1}^{\prime },b_{2}^{\prime },\cdots$, $b_{n-1}^{\prime }$ and $b_{n}^{\prime }$, where for i=1,2,⋯,n−1, $b_{i}^{\prime }$ contains $C_{\min}$ source symbols, and $b_{n}^{\prime }$ contains the remaining $L+z-\left(n-1\right)C_{\min}$ source symbols. Here, we note from (25) and (28) that

$$1\leq L+z-\left(n-1\right)C_{\min}\leq C_{\min}.$$

Thus, it suffices to construct, at most, 2 scalar-linear network codes of dimensions $C_{\min}$ and $\omega ≜L+z-\left(n-1\right)C_{\min}$, respectively, to multicast the L+z source symbols to all the sink nodes.

Let $C_{1}$ be a $C_{\min}$-dimensional scalar-linear network code in the network N, of which the global encoding vectors are column vectors ${\vec{f}}_{e}$ in $F^{C_{\min}}$ for all e∈E, and let $C_{2}$ be an ω-dimensional scalar-linear network code on N, of which the global encoding vectors are column vectors ${\vec{f}}_{e}^{\prime }$ in $F^{\omega }$ for all e∈E (cf. [1,2] and [6]). We use two codes $C_{1}$ and $C_{2}$ to construct an (L+z)-dimensional (vector-) linear network code C on the network N such that *n* symbols are transmitted on each edge e∈E. Specifically, for each e∈E, we let

$$\begin{array}{cc} G_{e} & =\left[{\vec{g}}_{e}^{\left(1\right)}\;{\vec{g}}_{e}^{\left(2\right)}\;\cdots \;{\vec{g}}_{e}^{\left(n\right)}\right]\\ \; & =\left[\begin{array}{ccccc} {\vec{f}}_{e} & \vec{0} & \cdots & \vec{0} & \vec{0}\\ \vec{0} & {\vec{f}}_{e} & \cdots & \vec{0} & \vec{0}\\ \vdots & \vdots & \ddots & \vdots & \vdots \\ \vec{0} & \vec{0} & \cdots & {\vec{f}}_{e} & \vec{0}\\ \vec{0} & \vec{0} & \cdots & \vec{0} & {\vec{f}}_{e}^{\prime } \end{array}\right], \end{array}$$

which is an *F*-valued (L+z)×n matrix regarded as the global encoding matrix for the code C.

Next, for an edge e∈E, we use $\langle G_{e}\rangle$ to denote the vector space spanned by the column vectors of the matrix $G_{e}$, i.e.,

$$\langle G_{e}\rangle ≜\langle {\vec{g}}_{e}^{\left(1\right)},{\vec{g}}_{e}^{\left(2\right)},\cdots ,{\vec{g}}_{e}^{\left(n\right)}\rangle .$$

Furthermore, for a wiretap set $W\in W_{r}$, we use $G_{W}$ to denote the (L+z)×nr matrix whose column vectors are the column vectors of $G_{e}$ for all the edges e∈W, i.e.,

$$G_{W}=\left[G_{e}:\;e\in W\right]=\left[{\vec{g}}_{e}^{\left(1\right)}\;\;{\vec{g}}_{e}^{\left(2\right)}\;\;\cdots \;\;{\vec{g}}_{e}^{\left(n\right)}:\;e\in W\right],$$

Then, similarly, we use $\langle G_{W}\rangle$ to denote the vector space spanned by the column vectors of the matrix $G_{W}$, i.e.,

$$\langle G_{W}\rangle ≜\langle {\vec{g}}_{e}^{\left(1\right)},{\vec{g}}_{e}^{\left(2\right)},\cdots ,{\vec{g}}_{e}^{\left(n\right)}:\;e\in W\rangle .$$

Hence, we readily see that

$$\begin{array}{c} \langle G_{W}\rangle =\sum_{e\in W}\langle G_{e}\rangle . \end{array}$$

Now, we claim that there exist *F*-valued column (L+z)-vectors ${\vec{u}}_{i},\;i=1,2,\cdots ,\tau L$ such that

(29) $$\langle {\vec{u}}_{i}:1\leq i\leq \tau L\rangle ⋂\langle G_{W}\rangle =\left\{\vec{0}\right\},\;\;\forall \;W\in W_{r}.$$

To show this, we prove by induction on 1≤j≤τL that if we have j−1 linearly independent column vectors ${\vec{u}}_{1},{\vec{u}}_{2},\cdots ,{\vec{u}}_{j-1}$ in $F^{L+z}$ such that

$$\langle {\vec{u}}_{i}:1\leq i\leq j-1\rangle ⋂\langle G_{W}\rangle =\left\{\vec{0}\right\},\;\;\forall \;W\in W_{r},$$

then we can choose a column vector ${\vec{u}}_{j}\in F^{L+z}\setminus \langle {\vec{u}}_{i}:1\leq i\leq j-1\rangle$ such that

$$\langle {\vec{u}}_{i}:1\leq i\leq j\rangle ⋂\langle G_{W}\rangle =\left\{\vec{0}\right\},\;\;\forall \;W\in W_{r},$$

provided that |F|>|E|r. We consider

$$\begin{array}{cc} \; & \left|F^{L+z}\;\setminus \;\underset{W\in W_{r}}{⋃}\langle G_{W},\;{\vec{u}}_{1},{\vec{u}}_{2},\cdots ,{\vec{u}}_{j-1}\rangle \right| \end{array}$$

(30) $$\begin{array}{cc} \; & \geq {\left|F\right|}^{L+z}-|W_{r}{\left|\cdot |F\right|}^{nr+j-1} \end{array}$$

(31) $$\begin{array}{cc} \; & \;\geq {\left|F\right|}^{nr+\tau L}-|W_{r}{\left|\cdot |F\right|}^{nr+\tau L-1} \end{array}$$

(32) $$\begin{array}{cc} \; & \;\;\geq {\left|F\right|}^{nr+\tau L-1}\;\cdot \;\left(\left|F|-\right|W_{r}|\right)>0, \end{array}$$

where the inequality (30) follows because

$$\begin{array}{cc} \dim\left(\langle G_{W},\;{\vec{u}}_{1},{\vec{u}}_{2},\cdots ,{\vec{u}}_{j-1}\rangle \right) & \leq \dim\left(\langle G_{W}\rangle \right)+j-1\\ \; & \leq n|W|+j-1=nr+j-1,\;\;\forall \;W\in W_{r}; \end{array}$$

inequality (31) follows from L+z≥nr+τL according to (25) and inequality (32) follows from

|F|>|E|r=|Wr|.

Thus, we have proven the existence of such vectors ${\vec{u}}_{i},\;1\leq i\leq \tau L$ that satisfy the condition (29).

With the vectors ${\vec{u}}_{i},\;1\leq i\leq \tau L$, we let *U* be an *F*-valued (L+z)×(L+z) invertible matrix such that ${\vec{u}}_{i},\;1\leq i\leq \tau L$ are the first τL column vectors of *U*. Furthermore, we consider an (L+z)×τL matrix

(33) $${\hat{M}}_{L}≜\left[\begin{array}{c} M_{L}\\ 0 \end{array}\right],$$

where 0 is the z×τL zero matrix. In particular, when z=0 (cf. (24)), ${\hat{M}}_{L}=M_{L}$. Recalling that $M_{L}$ has full column rank, we readily see that ${\hat{M}}_{L}$ also has full column rank. With the full-column-rank matrix ${\hat{M}}_{L}$, we let Γ be an *F*-valued (L+z)×(L+z) invertible matrix such that the column vectors of ${\hat{M}}_{L}$ are the first τL column vectors of Γ. Then, we define the matrix

(34) $$Q≜\Gamma \cdot U^{-1},$$

which is of size (L+z)×(L+z) and also invertible over *F*.

Now, we consider the transformation Q·C of the code C by the matrix *Q*, i.e., Q·C is an *F*-valued (L+z)-dimensional linear network code on the network N, of which all the global encoding matrices are

$$H_{e}≜Q\cdot G_{e},\;\forall \;e\in E,$$

(cf. the transformation of a scalar-linear network code in [6], Section 19.3.1 and [19], Theorem 2). Next, we show that $\hat{C}≜Q\cdot C$ is an admissible *F*-valued $\left(L,M_{L}\right)$ linear security network code for the security model $\left\{\left(L,M_{L}\right),r\right\}$ by verifying the decoding and security conditions.

**Remark 1.**  *We now discuss the computational complexity of our code construction. Our code construction consists of two parts: (i) constructing the two linear network codes $C_{1}$ and $C_{2}$ of different dimensions, which are used to multicast all the L+z symbols to the sink nodes; and (ii) constructing the transformation matrix Q, or equivalently, constructing the τL column (L+z)-vectors ${\vec{u}}_{i},\;1\leq i\leq \tau L$ that satisfy the condition (29). We analyze the complexity of the two parts as follows.*

- *The linear network codes $C_{1}$ and $C_{2}$ can be constructed in polynomial time (cf. [4,6,7]);*
- *To obtain the column (L+z)-vectors ${\vec{u}}_{i},\;1\leq i\leq \tau L$ that satisfy (29), we, in turn, choose τL vectors ${\vec{u}}_{i}$ as follows:*
$${\vec{u}}_{i}\in F^{L+z}\;\setminus \;\underset{W\in W_{r}}{⋃}\langle G_{W},\;{\vec{u}}_{1},{\vec{u}}_{2},\cdots ,{\vec{u}}_{i-1}\rangle .$$
*According to ([35], Lemma 11), the vectors ${\vec{u}}_{i},\;1\leq i\leq \tau L$ can be found in*
$$O\left(\tau L{\left(L+z\right)}^{3}|W_{r}\left|+\tau L\left(L+z\right)\right|W_{r}{|}^{2}\right).$$

*By combining the above analysis, our code construction can be implemented in polynomial time.*

▸ **Verification of the decoding condition**:

We continue to consider the output of the source (b,k), where $b\in F^{L}$ is the vector of source symbols, and $k\in F^{z}$ is the key. In using the code $\hat{C}$, the implementation of the global encoding matrices $H_{e},\;e\in E$ is equivalent to linearly transforming (bk) into x≜(bk)·Q, then using the code C to multicast x to all the sink nodes in *T*.

Since the vector x can be correctly decoded at each t∈T when applying the code C, (bk) can be also correctly decoded at each t∈T, as can the vector b of source symbols. Thus, we have verified the decoding condition.

▸ **Verification of the security condition**:

In order to verify the security condition (4), we need the next lemma, which plays a crucial role in our code construction. This lemma provides a necessary and sufficient condition for a linear security network code to satisfy the security condition (4). For an edge e∈E, $\langle H_{e}\rangle$ denotes the vector space spanned by the column vectors of $H_{e}$, i.e.,

$$\langle H_{e}\rangle ≜\langle {\vec{h}}_{e}^{\left(1\right)},{\vec{h}}_{e}^{\left(2\right)},\cdots ,{\vec{h}}_{e}^{\left(n\right)}\rangle .$$

Furthermore, for a wiretap set $W\in W_{r}$, we let $H_{W}$ be the (L+z)×nr matrix that contains all the column vectors of the global encoding matrices $H_{e}$ for all the edges e∈W, i.e.,

$$H_{W}=\left[H_{e}:\;e\in W\right]=\left[{\vec{h}}_{e}^{\left(1\right)}\;\;{\vec{h}}_{e}^{\left(2\right)}\;\;\cdots \;\;{\vec{h}}_{e}^{\left(n\right)}:\;e\in W\right].$$

We let

$$\langle H_{W}\rangle ≜\langle {\vec{h}}_{e}^{\left(1\right)},{\vec{h}}_{e}^{\left(2\right)},\cdots ,{\vec{h}}_{e}^{\left(n\right)}:\;e\in W\rangle$$

be the vector space spanned by the column vectors of $H_{W}$. Evidently,

$$\langle H_{W}\rangle =\sum_{e\in W}\langle H_{e}\rangle .$$

**Lemma 2.**  *For the security model $\left\{\left(L,M_{L}\right),r\right\}$ over a finite field F with $0<r<C_{\min}$, let $\hat{C}$ be an F-valued $\left(L,M_{L}\right)$ linear security network code, of which the global encoding matrices are (L+z)×n matrices $H_{e}=\left[{\vec{h}}_{e}^{\left(1\right)}\;{\vec{h}}_{e}^{\left(2\right)}\;\cdots \;{\vec{h}}_{e}^{\left(n\right)}\right],\;e\in E$. Then, for the code $\hat{C}$, the security condition (4) is satisfied if and only if*

(35) $$\langle {\hat{M}}_{L}\rangle ⋂\langle H_{W}\rangle =\left\{\vec{0}\right\},\;\;\forall \;W\in W_{r},$$

*where ${\hat{M}}_{L}=\left[\begin{array}{c} M_{L}\\ 0 \end{array}\right]$ is an (L+z)×τL matrix as defined in (33).*

**Proof.**  See Appendix B. □

Now, we start to verify the security condition for our code construction. Toward this end, according to Lemma 2, it suffices to verify (35). For the constructed $\left(L,M_{L}\right)$ linear security network code $\hat{C}$, we have

(36) $$\langle {\vec{u}}_{i}:\;1\leq i\leq \tau L\rangle ⋂\langle G_{W}\rangle =\left\{\vec{0}\right\},\;\;\forall \;W\in W_{r}$$

(cf. (29)). We recall (34) that $Q=\Gamma \cdot U^{-1}$ is an (L+z)×(L+z) invertible matrix. Then, according to (36), we immediately obtain

(37) $$\begin{array}{c} \langle Q\cdot {\vec{u}}_{i}:\;1\leq i\leq \tau L\rangle ⋂\langle Q\cdot G_{W}\rangle =\left\{\vec{0}\right\},\;\;\forall \;W\in W_{r}. \end{array}$$

We note that

(38) $$\begin{array}{c} H_{W}=\left[H_{e}:\;e\in W\right]=Q\;\cdot \;\left[G_{e}:\;e\in W\right]=Q\;\cdot \;G_{W},\;\;\forall \;W\in W_{r}. \end{array}$$

Furthermore, we write

$$\begin{array}{c} \left[{\vec{u}}_{1}\;\;{\vec{u}}_{2}\;\;\cdots \;\;{\vec{u}}_{\tau L}\right]=U\;\cdot \;\left[\begin{array}{c} I_{\tau L}\\ 0 \end{array}\right], \end{array}$$

where we recall that ${\vec{u}}_{i},\;1\leq i\leq \tau L$ are the first τL column vectors of *U*, $I_{\tau L}$ is the τL×τL identity matrix and 0 is the (L+z−τL)×τL zero matrix. Then, we can see that

$$\begin{array}{cc} Q\cdot \left[{\vec{u}}_{1}\;\;{\vec{u}}_{2}\;\;\cdots \;\;{\vec{u}}_{\tau L}\right] & =Q\cdot U\cdot \left[\begin{array}{c} I_{\tau L}\\ 0 \end{array}\right] \end{array}$$

(39) $$\begin{array}{cc} \; & =\Gamma \cdot U^{-1}\cdot U\cdot \left[\begin{array}{c} I_{\tau L}\\ 0 \end{array}\right]\\ \; & =\Gamma \cdot \left[\begin{array}{c} I_{\tau L}\\ 0 \end{array}\right] \end{array}$$

(40) $$\begin{array}{cc} \; & ={\hat{M}}_{L}, \end{array}$$

where (39) follows from $Q=\Gamma \cdot U^{-1}$ (cf. (34)), and (40) follows because the column vectors of ${\hat{M}}_{L}$ are the first τL column vectors of Γ. Combining (38) and (40) with (37), we immediately prove that

$$\begin{array}{c} \langle {\hat{M}}_{L}\rangle ⋂\langle H_{W}\rangle =\left\{\vec{0}\right\},\;\;\forall \;W\in W_{r}. \end{array}$$

Thus, according to Lemma 2, we have verified the security condition. Combining all the above, Theorem 3 has been proven.

### 3.4. An Example to Illustrate Our Code Construction

**Figure 1:**
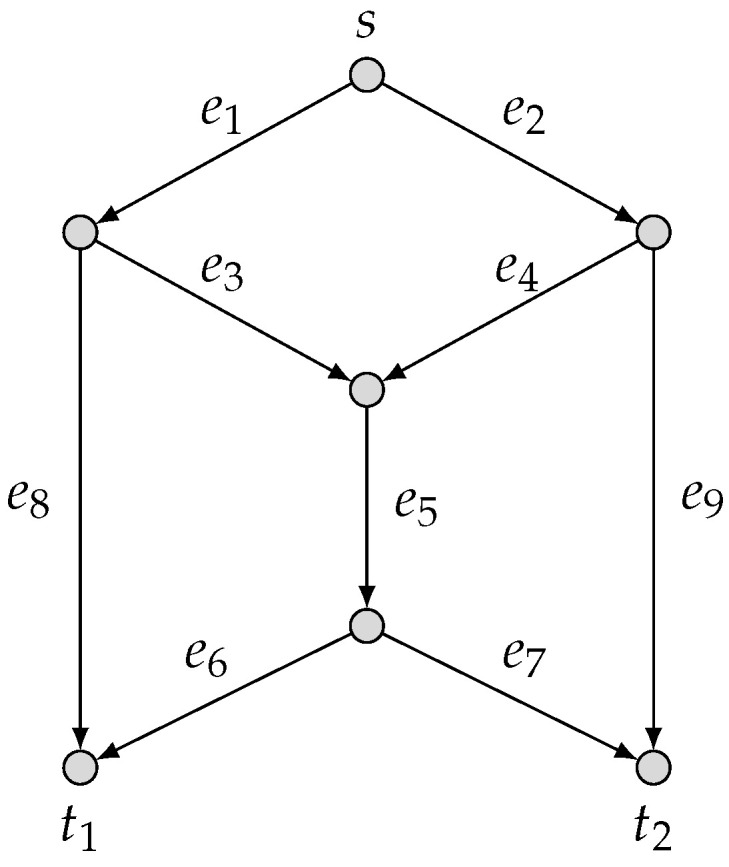
The butterfly network: $N=(G,s,T=\left\{t_{1},t_{2}\right\})$.

Let $N=(G,s,T=\left\{t_{1},t_{2}\right\})$ be the butterfly network as depicted in Figure 1. For the security model r=1, we consider two linear-combination security models $\left\{\left(2,M_{2}\right),1\right\}$ and $\left\{\left(3,M_{3}\right),1\right\}$ over the field $F_{3}=\left\{0,1,2\right\}$, where

(41) $$M_{2}=\left[\begin{array}{c} 1\\ 1 \end{array}\right]\;\;\mathrm{and}\;\;M_{3}=\left[\begin{array}{cc} 1 & 0\\ 1 & 1\\ 0 & 1 \end{array}\right].$$

Namely, in the $\left\{\left(2,M_{2}\right),1\right\}$ security model, the algebraic sum $B_{1}+B_{2}$ of the two source symbols is required to be protected from the wiretapper, and in the $\left\{\left(3,M_{3}\right),1\right\}$ security model, the algebraic sums $B_{1}+B_{2}$ and $B_{2}+B_{3}$ of the source symbols are required to be protected from the wiretapper.

- The security model: $\left\{\left(2,M_{2}\right),1\right\}$.

In this model, the source node *s* generates two source symbols $b_{1}$ and $b_{2}$ in $F_{3}$, and the algebraic sum $b_{1}+b_{2}$ needs to be protected. According to (41), we have

$$m_{2}=\mathrm{Rank}\left(M_{2}\right)=1,\;\;\mathrm{and}\;\;\tau =\frac{m_{2}}{2}=\frac{1}{2}=\tau_{0}=\frac{C_{\min}-r}{C_{\min}}.$$

Therefore, we have $0\leq \tau \leq \tau_{0}$, i.e., the first case in Theorem 2. Next, we construct an optimal $F_{3}$-valued $\left(2,M_{2}\right)$ linear security network code for the $\left\{\left(2,M_{2}\right),1\right\}$ security model, which achieves a security capacity of 2.

According to our code construction, it follows from (23) and (24) that we take

$$n=\left\lceil \frac{L}{C_{\min}}\right\rceil =1$$

and z=0 because L=2≥nr+τL=2. We first consider an $F_{3}$-valued two-dimensional scalar-linear network code $C_{1}$ on the network N, which is used to multicast two source symbols $b_{1}$ and $b_{2}$ in $F_{3}$ to sink nodes $t_{1}$ and $t_{2}$. The global encoding matrices (vectors) of $C_{1}$ are

$$\begin{array}{c} \begin{array}{c} G_{e_{1}}=G_{e_{3}}=G_{e_{8}}=\left[\begin{array}{c} 1\\ 0 \end{array}\right],\;G_{e_{2}}=G_{e_{4}}=G_{e_{9}}=\left[\begin{array}{c} 0\\ 1 \end{array}\right],\;\;\mathrm{and}\;\;G_{e_{5}}=G_{e_{6}}=G_{e_{7}}=\left[\begin{array}{c} 1\\ 1 \end{array}\right]. \end{array} \end{array}$$

Clearly, the code $C_{1}$ is not secure for the algebraic sum $b_{1}+b_{2}$ because the wiretapper can obtain $b_{1}+b_{2}$ by accessing the edge $e_{5}$ on which $b_{1}+b_{2}$ is transmitted. Based on the code $C_{1}$, we now construct a $\left(2,M_{2}\right)$ scalar-linear security network code for the $\left\{\left(2,M_{2}\right),1\right\}$ security model.

Next, we let ${\vec{u}}_{1}=\left[\begin{array}{c} 1\\ 2 \end{array}\right]$, an $F_{3}$-valued column 2-vector such that ${\vec{u}}_{1}\notin \langle G_{e_{i}}\rangle ,\;\forall \;1\leq i\leq 9$ (cf. (29)). Then, let $U=\left[\begin{array}{cc} 1 & 0\\ 2 & 1 \end{array}\right]$, a 2×2 invertible matrix on $F_{3}$ such that ${\vec{u}}_{1}$ is the first column vector of *U*. Furthermore, since z=0, we have ${\hat{M}}_{2}=M_{2}=\left[\begin{array}{c} 1\\ 1 \end{array}\right]$ (cf. (33)) and let $\Gamma =\left[\begin{array}{cc} 1 & 0\\ 1 & 1 \end{array}\right]$, which is a 2×2 invertible matrix on $F_{3}$ such that $\left[\begin{array}{c} 1\\ 1 \end{array}\right]$ is the first column vector of Γ. According to (34), we calculate $Q=\Gamma \cdot U^{-1}=\left[\begin{array}{cc} 1 & 0\\ 2 & 1 \end{array}\right]$. Now, we obtain an admissible $F_{3}$-valued $\left(2,M_{2}\right)$ scalar-linear security network code ${\hat{C}}_{1}=Q\cdot C_{1}$, of which the global encoding matrices (vectors) are $H_{e_{i}}=Q\cdot G_{e_{i}},\;1\leq i\leq 9$. Specifically,

$$\begin{array}{c} \begin{array}{c} H_{e_{1}}=H_{e_{3}}=H_{e_{8}}=\left[\begin{array}{c} 1\\ 2 \end{array}\right],\;H_{e_{2}}=H_{e_{4}}=H_{e_{9}}=\left[\begin{array}{c} 0\\ 1 \end{array}\right],\;\;\mathrm{and}\;\;H_{e_{5}}=H_{e_{6}}=H_{e_{7}}=\left[\begin{array}{c} 1\\ 0 \end{array}\right]. \end{array} \end{array}$$

**Figure 2:**
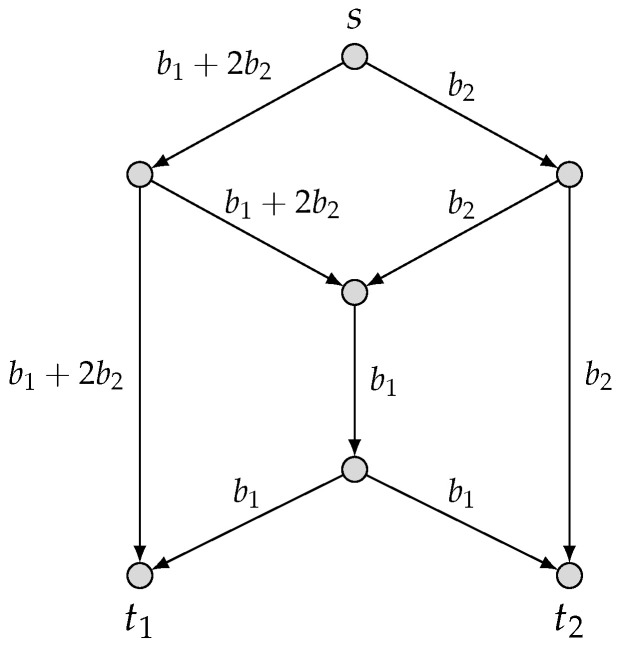
An $F_{3}$-valued $\left(2,M_{2}\right)$ scalar-linear security network code for $\left\{\left(2,M_{2}\right),1\right\}$.

We use $y_{e_{i}}$, which takes values in $F_{3}$, to denote the message transmitted on each edge $e_{i},\;1\leq i\leq 9$. According to the above global encoding matrices of ${\hat{C}}_{1}$, the messages $y_{e_{i}}\;\left(=\left(b_{1},b_{2}\right)\cdot H_{e}\right)$ transmitted on the edges $e_{i},\;1\leq i\leq 9$ are

$$\begin{array}{c} \begin{array}{c} y_{e_{1}}=y_{e_{3}}=y_{e_{8}}=b_{1}+2b_{2},\;y_{e_{2}}=y_{e_{4}}=y_{e_{9}}=b_{2},\;\;\mathrm{and}\;\;y_{e_{5}}=y_{e_{6}}=y_{e_{7}}=b_{1}, \end{array} \end{array}$$

as depicted in Figure 2. We can readily verify the decoding and security conditions for the code ${\hat{C}}_{1}$. In particular, in this case, we see that although no randomness is used to randomize the source symbols, the wiretapper cannot obtain any information about the algebraic sum $b_{1}+b_{2}$ when any one edge is eavesdropped.

- The security model: $\left\{\left(3,M_{3}\right),1\right\}$.

In this model, the source node *s* generates three source symbols $b_{1},b_{2}$ and $b_{3}$ in $F_{3}$, and two algebraic sums $b_{1}+b_{2}$ and $b_{2}+b_{3}$ need to be protected. According to (41), we note that $m_{3}=\mathrm{Rank}\left(M_{3}\right)=2$; thus,

$$\tau =\frac{m_{3}}{3}=\frac{2}{3}>\tau_{0}=\frac{C_{\min}-r}{C_{\min}}=\frac{1}{2}.$$

Therefore, we have $\tau_{0}<\tau \leq 1$, i.e., the second case in Theorem 2. Next, we construct an optimal $F_{3}$-valued $\left(3,M_{3}\right)$ linear security network code for the $\left\{\left(3,M_{3}\right),1\right\}$ security model, which achieves a security capacity of 3/2.

According to our code construction, it follows from (23) and (24) that we take

$$n=\left\lceil \frac{\tau L}{C_{\min}-r}\right\rceil =2$$

and z=1 because L<nr+τL according to L=3 and nr+τL=4. We consider an $F_{3}$-valued four-dimensional (where 4=L+z) linear network code $C_{2}$ of rate 2, which is used to multicast the three source symbols $b_{1}$, $b_{2}$ and $b_{3}$ and a key *k* in $F_{3}$ to the sink nodes $t_{1}$ and $t_{2}$. The 4×2 global encoding matrices of $C_{2}$ are

$$\begin{array}{c} \begin{array}{c} G_{e_{1}}\;=\;G_{e_{3}}\;=\;G_{e_{8}}\;=\;\left[\begin{array}{cc} 1 & 0\\ 0 & 0\\ 0 & 1\\ 0 & 0 \end{array}\right],\;G_{e_{2}}\;=\;G_{e_{4}}\;=\;G_{e_{9}}\;=\;\left[\begin{array}{cc} 0 & 0\\ 1 & 0\\ 0 & 0\\ 0 & 1 \end{array}\right],\;\mathrm{and}\;G_{e_{5}}\;=\;G_{e_{6}}\;=\;G_{e_{7}}\;=\;\left[\begin{array}{cc} 1 & 0\\ 1 & 0\\ 0 & 1\\ 0 & 1 \end{array}\right]. \end{array} \end{array}$$

We note that the code $C_{2}$ is not secure because the wiretapper can obtain some information about $b_{1}+b_{2}$ by accessing the edge $e_{5}$ on which $b_{1}+b_{2}$ and $b_{3}+k$ are transmitted. Based on the code $C_{2}$, we now construct a linear secure network code for the $\left\{\left(3,M_{3}\right),1\right\}$ security model.

Let

$${\vec{u}}_{1}=\left[\begin{array}{c} 1\\ 2\\ 0\\ 0 \end{array}\right]\;\;\mathrm{and}\;\;{\vec{u}}_{2}=\left[\begin{array}{c} 0\\ 0\\ 1\\ 2 \end{array}\right]$$

be two $F_{3}$-valued column 4-vectors such that $\langle {\vec{u}}_{1},{\vec{u}}_{2}\rangle ⋂\langle G_{e_{i}}\rangle =\left\{\vec{0}\right\},\;\forall \;1\leq i\leq 9$ (cf. (29)). Then, let

$$U=\left[\begin{array}{cccc} 1 & 0 & 0 & 0\\ 2 & 0 & 1 & 0\\ 0 & 1 & 0 & 0\\ 0 & 2 & 0 & 1 \end{array}\right]$$

be a 4×4 invertible matrix on $F_{3}$ such that ${\vec{u}}_{1}$ and ${\vec{u}}_{2}$ are the first two column vectors of *U*. Furthermore, since z=1, as mentioned above, we have

$${\hat{M}}_{3}=\left[\begin{array}{cc} 1 & 0\\ 1 & 1\\ 0 & 1\\ 0 & 0 \end{array}\right]$$

(cf. (33)). Also let

$$\Gamma =\left[\begin{array}{cccc} 1 & 0 & 0 & 0\\ 1 & 1 & 0 & 0\\ 0 & 1 & 1 & 0\\ 0 & 0 & 0 & 1 \end{array}\right]$$

be a 4×4 invertible matrix on $F_{3}$ such that the column vectors of ${\hat{M}}_{L}$ are the first two column vectors of Γ. According to (34), we calculate

$$Q=\Gamma \cdot U^{-1}=\left[\begin{array}{cccc} 1 & 0 & 0 & 0\\ 1 & 0 & 1 & 0\\ 1 & 1 & 1 & 0\\ 0 & 0 & 1 & 1 \end{array}\right],$$

Now, we obtain an admissible $F_{3}$-valued $\left(3,M_{3}\right)$ linear security network code ${\hat{C}}_{2}=Q\cdot C_{2}$, of which the 4×2 global encoding matrices are $H_{e_{i}}=Q\cdot G_{e_{i}},\;1\leq i\leq 9$; specifically,

$$\begin{array}{c} \begin{array}{c} H_{e_{1}}\;=\;H_{e_{3}}\;=\;H_{e_{8}}\;=\;\left[\begin{array}{cc} 1 & 0\\ 1 & 1\\ 1 & 1\\ 0 & 1 \end{array}\right],\;H_{e_{2}}\;=\;H_{e_{4}}\;=\;H_{e_{9}}\;=\;\left[\begin{array}{cc} 0 & 0\\ 0 & 0\\ 1 & 0\\ 0 & 1 \end{array}\right],\;\mathrm{and}\;H_{e_{5}}\;=\;H_{e_{6}}\;=\;H_{e_{7}}\;=\;\left[\begin{array}{cc} 1 & 0\\ 1 & 1\\ 2 & 1\\ 0 & 2 \end{array}\right]. \end{array} \end{array}$$

**Figure 3:**
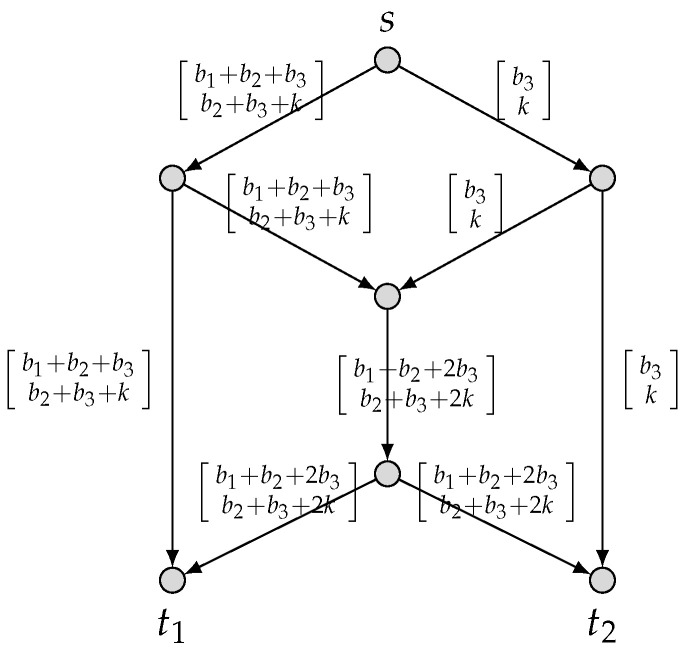
An $F_{3}$-valued $\left(3,M_{3}\right)$ linear-security network code for $\left\{\left(3,M_{3}\right),1\right\}$.

We use $y_{e_{i}}$, which takes values in $F_{3}^{2}$, to denote the message transmitted on each edge $e_{i},\;1\leq i\leq 9$. According to the above global encoding matrices of ${\hat{C}}_{2}$, the messages $y_{e_{i}}\;\left(=\left(b_{1},b_{2},b_{3},k\right)\cdot H_{e}\right)$ transmitted on the edges $e_{i},\;1\leq i\leq 9$ are

$$\begin{array}{cc} \; & y_{e_{1}}=y_{e_{3}}=y_{e_{8}}=\left(b_{1}+b_{2}+b_{3},\;b_{2}+b_{3}+k\right),\\ y_{e_{2}}=y_{e_{4}} & =y_{e_{9}}=\left(b_{3},\;k\right),\;\;\mathrm{and}\;\;y_{e_{5}}=y_{e_{6}}=y_{e_{7}}=\left(b_{1}+b_{2}+2b_{3},\;b_{2}+b_{3}+2k\right), \end{array}$$

as depicted in Figure 3.

For the $\left\{\left(2,M_{2}\right),1\right\}$ and $\left\{\left(3,M_{3}\right),1\right\}$ security models, as discussed in the above example, according to Theorem 3, admissible linear security network codes with rates of 2 and 3/2, respectively, can be constructed if the field size is |F|>max|T|,|E|r=9. However, we see in the example that the field $F_{3}$, of size 3 is sufficient for our code construction. This implies that the max|T|,|E|r bound in Theorem 3 on the field size is only sufficient but not necessary for our code construction.

## 4. Asymptotic Behavior of the Security Capacity

In this section, we investigate the asymptotic behavior of the security capacity. For a fixed network N and a security level *r*, we consider a sequence of the $\{\left(L,M_{L}\right),r\},\;L=1,2,\cdots$ security models. The following theorem characterizes the asymptotic behavior of the security capacity for a sequence of security models $\{\left(L,M_{L}\right),r\},\;L=1,2,\cdots$.

**Theorem 4.**  *Consider a sequence of linear-combination security models $\left\{\left(L,M_{L}\right),r\right\}$ over a finite field F for L=1,2,⋯, where $0<r<C_{\min}$ and |F|>max|T|,|E|r. $C_{L,M_{L}}$ denotes the security capacity for each model $\left\{\left(L,M_{L}\right),r\right\}$. Let*

$$\tau_{L}=\frac{m_{L}}{L},\;L=1,2,\cdots \;\;\mathrm{and}\;\;\tau_{0}=\frac{C_{\min}-r}{C_{\min}},$$

*where $m_{L}=\mathrm{Rank}\left(M_{L}\right)$ for L=1,2,⋯.*

- *If $\tau_{L}\leq \tau_{0}+o\left(1\right)$, then,*
$$\lim_{L\to \infty }C_{L,M_{L}}=C_{\min}.$$
- *If $\tau_{L}=\kappa +o\left(1\right)$, with κ satisfying $\tau_{0}<\kappa \leq 1$, then,*
$$\lim_{L\to \infty }C_{L,M_{L}}=\kappa^{-1}\cdot \left(C_{\min}-r\right).$$

**Proof.**  We first consider the case of $\tau_{L}\leq \tau_{0}+o\left(1\right)$. Then, there exists a non-negative sequence, $a_{L},\;L=1,2,\cdots$ with $\lim_{L\to \infty }a_{L}=0$, such that

(42) $$\tau_{L}\leq \tau_{0}+a_{L},\;L=1,2,\cdots .$$

We now use Theorem 2 to show that

(43) $$C_{L,M_{L}}\geq \frac{L}{\left\lceil \left(\tau_{0}+a_{L}\right)\cdot L/\left(C_{\min}-r\right)\right\rceil }.$$

To show this, consider the following two cases:

- If $0\leq \tau_{L}\leq \tau_{0}$, it follows from (19) that
  $$C_{L,M_{L}}=\frac{L}{\left\lceil L/C_{\min}\right\rceil }=\frac{L}{\left\lceil \tau_{0}\;\cdot \;L/\left(C_{\min}-r\right)\right\rceil }\geq \frac{L}{\left\lceil \left(\tau_{0}+a_{L}\right)\cdot L/\left(C_{\min}-r\right)\right\rceil };$$
- If $\tau_{0}<\tau_{L}\leq 1$, then we obtain
  $$C_{L,M_{L}}=\frac{L}{\left\lceil \tau_{L}\cdot L/\left(C_{\min}-r\right)\right\rceil }\geq \frac{L}{\left\lceil \left(\tau_{0}+a_{L}\right)\cdot L/\left(C_{\min}-r\right)\right\rceil },$$
  where the equality follows from (20), and the inequality follows from (42).

Combining (43) and (7) with Lemma 1, we further obtain that for each pair $\left(L,M_{L}\right)$,

(44) $$\frac{L}{\left\lceil \left(\tau_{0}+a_{L}\right)\cdot L/\left(C_{\min}-r\right)\right\rceil }\leq C_{L,M_{L}}\leq \frac{L}{\left\lceil L/C_{\min}\right\rceil }\leq C_{\min}.$$

We note that

$$\lim_{L\to \infty }\frac{L}{\left\lceil \left(\tau_{0}+a_{L}\right)\cdot L/\left(C_{\min}-r\right)\right\rceil }=C_{\min}.$$

Together with (44), we have thus proven that

$$\lim_{L\to \infty }C_{L,M_{L}}=C_{\min}.$$

Next, we consider a case in which $\tau_{L}=\kappa +o\left(1\right)$, where $\tau_{0}<\kappa \leq 1$. Then, there exists a sequence $b_{L}$, L=1,2,⋯ satisfying $\lim_{L\to \infty }b_{L}=0$ such that

$$\tau_{L}=\kappa +b_{L},\;L=1,2,\cdots .$$

Here, we note that $b_{L}$ may be negative. Together with $\kappa >\tau_{0}$ and $\lim_{L\to \infty }b_{L}=0$, there exists a positive integer $L_{0}$ such that for each $L\geq L_{0}$,

$$|b_{L}|<\kappa -\tau_{0},\;\;i.e.,\;\;\tau_{0}-\kappa <b_{L}<\kappa -\tau_{0},$$

which implies that

$$\tau_{L}=\kappa +b_{L}>\tau_{0},\;\forall \;L\geq L_{0}.$$

According to (20) in Theorem 2, we have

$$C_{L,M_{L}}=\frac{L}{\left\lceil \left(\kappa +b_{L}\right)\cdot L/\left(C_{\min}-r\right)\right\rceil },$$

so that

$$\lim_{L\to \infty }C_{L,M_{L}}=\kappa^{-1}\cdot \left(C_{\min}-r\right).$$

Thus, the theorem is proven. □

According to Theorem 4, we can see that for a sequence of security models $\{\left(L,M_{L}\right),r\},\;L=1,2,\cdots$ that satisfies $\tau_{L}\leq \tau_{0}+o\left(1\right)$ or $\tau_{L}=\kappa +o\left(1\right)$, where $\tau_{0}<\kappa \leq 1$, our code construction is *asymptotically optimal*, i.e.,

(45) $$\lim_{L\to \infty }R\left({\hat{C}}_{L,M_{L}}\right)=\lim_{L\to \infty }C_{L,M_{L}},$$

where ${\hat{C}}_{L,M_{L}}$ is the code constructed for each model $\left\{\left(L,M_{L}\right),r\right\}$ by our code construction. To illustrate this, in the following, we consider several specific sequences of security models.

First, we consider a sequence of security models $\{\left(L,M_{L}\right),r\},\;L=1,2,\cdots$ in which all the ranks $\mathrm{Rank}(M_{L}),\;L=1,2,\cdots$ are upper-bounded by a constant, such as *m*, e.g., the security constraint of multiple algebraic sums,

∑i∈[L]:i≡j(modm)Bi,j=1,2,⋯,m

as discussed in the last paragraph of Section 2. With this, we have

$$\lim_{L\to \infty }\frac{m_{L}}{L}=0,$$

which implies the inequality $\tau_{L}=m_{L}/L\leq \tau_{0}+o\left(1\right)$. It then follows from the first case of Theorem 4 that

$$\lim_{L\to \infty }C_{L,M_{L}}=C_{\min}.$$

Next, we show that our code construction is asymptotically optimal. We first note that

$$\tau_{L}=\frac{m_{L}}{L}\leq \frac{C_{\min}-r}{C_{\min}}=\tau_{0},\;\forall \;L\geq C_{\min}\cdot \left\lceil \frac{m}{C_{\min}-r}\right\rceil .$$

Together with the first case of Theorem 3 (cf. (21)), the constructed code ${\hat{C}}_{L,M_{L}}$ achieves a rate of $R\left({\hat{C}}_{L,M_{L}}\right)=\frac{L}{\left\lceil L/C_{\min}\right\rceil }$. This immediately implies that the equality (45) is satisfied, namely that our code construction is asymptotically optimal for this example.

Next, we consider a sequence of security models $\{\left(L,M_{L}\right),r\},\;L=1,2,\cdots$ in which all the ranks $m_{L}=\mathrm{Rank}\left(M_{L}\right)$ satisfy

$$m_{L}=\left\lceil \kappa \cdot L\right\rceil ,\;\;L=1,2,\cdots .$$

We note that the sequence of $m_{L},\;L=1,2,\cdots$ is not upper-bounded. According to Theorem 4, we can obtain the asymptotic behavior of the security capacity for the sequence of models $\{\left(L,M_{L}\right),r\},\;L=1,2,\cdots$ as follows:

(46) $$\lim_{L\to \infty }C_{L,M_{L}}=\left\{\begin{array}{cc} C_{\min}, & \mathrm{if}0<\kappa \leq \tau_{0},\\ \kappa^{-1}\cdot \left(C_{\min}-r\right), & \mathrm{if}\tau_{0}<\kappa <1. \end{array}\right.$$

Furthermore, it follows from Theorem 3 that

(47) $$\lim_{L\to \infty }R\left({\hat{C}}_{L,M_{L}}\right)=\left\{\begin{array}{cc} C_{\min}, & \mathrm{if}0<\kappa \leq \tau_{0},\\ \kappa^{-1}\cdot \left(C_{\min}-r\right), & \mathrm{if}\tau_{0}<\kappa <1, \end{array}\right.$$

where ${\hat{C}}_{L,M_{L}}$ is the code constructed for each model $\left\{\left(L,M_{L}\right),r\right\}$ by the code construction. Comparing (46) and (47), we immediately see that the equality (45) holds, which shows that our code construction is asymptotically optimal for this example.

Finally, we consider the special sequence of security models $\left\{\left(L,M_{L}\right),r\right\}$ for L=1,2,⋯, where $m_{L}=L$, i.e., $\tau_{L}=m_{L}/L=1$ for all L=1,2,⋯. This linear-combination security constraint is equivalent to protecting all the source symbols from the wiretapper, so each model $\left\{\left(L,M_{L}\right),r\right\}$ is equivalent to the standard secure-network coding model. Thus, we have

(48) $$\lim_{L\to \infty }C_{L,M_{L}}=C_{\min}-r.$$

On the other hand, for each pair $\left(L,M_{L}\right)$, it follows from $\tau_{L}=1$ and Theorem 3 that the $\left(L,M_{L}\right)$ linear security network code ${\hat{C}}_{L,M_{L}}$ constructed by our code construction has a rate of

$$R\left({\hat{C}}_{L,M_{L}}\right)=\frac{L}{\left\lceil L/(C_{\min}-r)\right\rceil }.$$

This implies that

(49) $$\lim_{L\to \infty }R\left({\hat{C}}_{L,M_{L}}\right)=C_{\min}-r.$$

Combining (48) and (49), we see that the equality (45) holds, and thus, our code construction is also asymptotically optimal for this example.

## 5. Conclusions

In this paper, we put forward the model of multiple linear-combination security network coding, which is specified by the security level, the number of source symbols and the linear-combination security constraint. We fully characterized the security capacity for any such security model in terms of the ratio τ of the rank of the linear-combination security constraint to the number of source symbols. Also, we developed a construction of linear security network codes. The code construction is applicable to any security model, and the constructed code achieves the security capacity. We also determined a threshold value $\tau_{0}$ such that there is no penalty on the security capacity compared with the capacity without any security consideration when the ratio τ is not larger than $\tau_{0}$. Finally, we analyzed the asymptotic behavior of the security capacity for a sequence of linear-combination security models and fully characterized the asymptotic behavior of the security capacity. We also showed that our code construction is asymptotically optimal.

## Data Availability

Not applicable.

## Appendix A. A Related Work by Bhattad and Narayanan

A model related to the current work is that considered by Bhattad and Narayanan [23], the general case of which is given as follows. On the network N, the single source node *s* generates *L* $L\leq C_{\min}$ source symbols, as denoted by $X_{1},X_{2},\cdots ,X_{L}$, over a finite field *F*, which are required to be multicast to all the sink nodes in *T*. Let $U_{p},\;1\leq p\leq P$ be *P* subsets of the *L* source symbols and $G_{p},$1≤p≤P be another *P* subsets of the *L* source symbols. The security requirement is specified by the *P* pairs $(U_{p},G_{p}),\;1\leq p\leq P$ as follows. The wiretapper, who can access any one wiretap set *W* in a collection W of wiretap sets, is not allowed to obtain any information about $U_{p}$, given $G_{p}$ for each p=1,2,⋯,P, i.e., for each p=1,2,⋯,P,

(A1) $$I\left(U_{p};Y_{W}|G_{p}\right)=0\;\mathrm{or}\;H\left(U_{p}|G_{p}\right)=H\left(U_{p}|Y_{W},G_{p}\right),\;\forall \;W\in W,$$

where $Y_{W}=\left(Y_{e}:\;e\in W\right)$, with $Y_{e}$ being the random variable transmitted on the edge *e*. In particular, when taking P=L, $U_{p}=\left\{X_{p}\right\}$ and $G_{p}=\emptyset$ for 1≤p≤P, the security requirement (A1) becomes

$$I\left(X_{p};Y_{W}\right)=0,\;\;\forall \;1\leq p\leq P\;\;\mathrm{and}\;\;W\in W.$$

This type of security requirement is called *weak security* in [23].

For the above model, the main focus in [23] is on how to find a suitable linear transformation of the *L* source symbols for a given linear network code to obtain a secure linear network code such that the security requirement (A1) is satisfied. Theorem 3 in [23], the most general result presented in the paper, asserts the existence of such a linear transformation when a given condition is satisfied. We state this theorem as follows.

**Theorem A1** ([23], Theorem 3). *Consider an L-dimensional $L\leq C_{\min}$ network code C over a finite field F and a collection of wiretap sets W in which $r=\max_{W\in W}\left|W\right|$. Let $(U_{p},G_{p}),\;1\leq p\leq P$ be P pairs of subsets $U_{p}$ and $G_{p}$ of the L source symbols, which specify the security requirement. If*

(A2) $$\max_{1\leq p\leq P}\left(|U_{p}\left|+\right|G_{p}|\right)\leq L-r,$$

*then there exists a linear transformation of the source symbols as a precoding on the linear network code C such that the security requirement (A1) is satisfied.*

We now go back to the linear-combination security model $\left\{\left(L,M_{L}\right),r\right\}$ discussed in the current paper. Consider the first case $0\leq \tau \leq \tau_{0}$ in Theorem 2, where we recall that $\tau =m_{L}/L$, where $m_{L}=\mathrm{Rank}\left(M_{L}\right)$ and $\tau_{0}=\left(C_{\min}-r\right)/C_{\min}$. Then, we can apply the approach of the linear transformation in Theorem A1 (cf. [23] for details) to obtain a $\left(L,M_{L}\right)$ linear security network code, provided that the following two additional conditions on the model parameters are satisfied:

(A3) $$0\leq \tau \leq \frac{L-r}{L}\;\left(\leq \tau_{0}\right)\;\mathrm{and}\;L\leq C_{\min}.$$

Specifically, we consider a linear-combination security model $\left\{\left(L,M_{L}\right),r\right\}$ satisfying the conditions (A3), where the *L* source symbols $B_{1},B_{2},\cdots ,B_{L}$ are required to be multicast to all the sink nodes in *T*, and the multiple linear combinations $B\cdot M_{L}$, where $B=(B_{1},B_{2},\cdots ,B_{L})$ are required to be protected from the wiretapper. We first linearly transform $B=(B_{1},B_{2},\cdots ,B_{L})$ to $\left(X_{1},X_{2},\cdots ,X_{L}\right)$ using an L×L invertible matrix *M* whose left $L\times m_{L}$ submatrix is equal to $M_{L}$. Then, we have

$$\left(X_{1},X_{2},\cdots ,X_{L}\right)=\left(B_{1},B_{2},\cdots ,B_{L}\right)\cdot M,$$

where

$$\left(X_{1},X_{2},\cdots ,X_{m_{L}}\right)=\left(B_{1},B_{2},\cdots ,B_{L}\right)\cdot M_{L}.$$

We now apply Theorem A1 as follows. Take $X_{1},X_{2},\cdots ,X_{L}$ as the source symbols. Let $U=\{X_{1},X_{2},\cdots ,X_{m_{L}}\}$, G=∅ and $W=W_{r}$. According to τ≤(L−r)/L, we see that $\left|U\right|+\left|G\right|=m_{L}\leq L-r$, which satisfies the condition (A2) in Theorem A1. It therefore follows from Theorem A1 that we can construct a linear secure network code such that $X_{1},X_{2},\cdots ,X_{L}$ can be multicast to all the sink nodes in *T*, and the wiretapper cannot obtain any information about *U*, i.e.,

$$I\left(X_{1},X_{2},\cdots ,X_{m_{L}};Y_{W}\right)=0,\;\;\forall \;W\in W_{r},$$

or, equivalently,

$$I\left(B\cdot M_{L};Y_{W}\right)=0,\;\;\forall \;W\in W_{r}.$$

Hence, we obtain an admissible $\left(L,M_{L}\right)$ linear security network code for the security model $\left\{\left(L,M_{L}\right),r\right\}$.

However, the second case $\tau_{0}<\tau \leq 1$ in Theorem 2 cannot be handled by the approach proposed in [23]. Specifically, according to $\tau >\tau_{0}$, we have

$$\frac{m_{L}}{L}=\tau >\tau_{0}=\frac{C_{\min}-r}{C_{\min}}\geq \frac{L-r}{L}.$$

This implies that $\left|U\right|+\left|G\right|=m_{L}>L-r$, which does not satisfy the condition (A2) in Theorem A1. Hence, we cannot apply the linear transformation approach for the case of $\tau_{0}<\tau \leq 1$.

## Appendix B. Proof of Lemma 2

We first prove the “only if” part by contradiction. Suppose, on the contrary, that there exists a wiretap set $W\in W_{r}$ such that

(A4) $$\begin{array}{c} \langle {\hat{M}}_{L}\rangle ⋂\langle H_{W}\rangle \neq \left\{\vec{0}\right\}. \end{array}$$

In the following, we prove that

(A5) $$I\left(B\;\cdot \;M_{L};\;Y_{W}\right)>0,$$

which contradicts the security condition (4) for the code $\hat{C}$.

According (A4), there exist two non-zero column vectors $\vec{w}\in F^{n|W|}$ ($=F^{nr}$) and $\vec{u}\in F^{\tau L}$ such that

(A6) $$H_{W}\cdot \vec{w}={\hat{M}}_{L}\cdot \vec{u}\neq \vec{0},$$

where $\vec{0}$ is the zero-column (L+z)-vector. Then, we obtain

(A7) $$\begin{array}{cc} \; & \;\;\;\;\;\;I\left(B\;\cdot \;M_{L};\;Y_{W}\right)\\ \; & \;\;\;\;\;\;=I\left(B\;\cdot \;M_{L};\;\left(B\;K\right)\;\cdot \;H_{W}\right)\\ \; & \;\;\;\;\;\;=H\left(B\;\cdot \;M_{L}\right)-H\left(B\;\cdot \;M_{L}|\left(B\;K\right)\;\cdot \;H_{W}\right) \end{array}$$

(A8) $$\begin{array}{cc} \; & \;\;\;\;\;\;\;\;\;\;\;\;=H\left(B\;\cdot \;M_{L}\right)-H\left(B\;\cdot \;M_{L}\;\cdot \;|\left(B\;K\right)\;\cdot \;H_{W},\left(B\;K\right)\;\cdot \;H_{W}\;\cdot \;\vec{w}\right)\\ \; & \;\;\;\;\;\;\;\;\;\;\;\;\geq H\left(B\;\cdot \;M_{L}\right)-H\left(B\;\cdot \;M_{L}|\left(B\;K\right)\;\cdot \;H_{W}\;\cdot \;\vec{w}\right)\\ \; & \;\;\;\;\;\;\;\;\;\;\;\;=I\left(B\;\cdot \;M_{L};\;\left(B\;K\right)\;\cdot \;H_{W}\;\cdot \;\vec{w}\right)\\ \; & \;\;\;\;\;\;\;\;\;\;\;\;=I\left(B\;\cdot \;M_{L};\;\left(B\;K\right)\;\cdot \;{\hat{M}}_{L}\;\cdot \;\vec{u}\right)\\ \; & \;\;\;\;\;\;\;\;\;\;\;\;=I\left(B\;\cdot \;M_{L};\;B\;\cdot \;M_{L}\;\cdot \;\vec{u}\right)\\ \; & \;\;\;\;\;\;\;\;\;\;\;\;=H\left(B\;\cdot \;M_{L}\;\cdot \;\vec{u}\right)-H\left(B\;\cdot \;M_{L}\;\cdot \;\vec{u}|B\;\cdot \;M_{L}\right) \end{array}$$

(A9) $$\begin{array}{cc} \; & =H\left(B\;\cdot \;M_{L}\;\cdot \;\vec{u}\right)>0, \end{array}$$

where the equality in (A7) follows from $Y_{W}=\left(B\;K\right)\cdot H_{W}$, the equality in (A8) follows from (A6), the equality in (A9) follows from $H\left(B\;\cdot \;M_{L}\;\cdot \;\vec{u}|B\;\cdot \;M_{L}\right)=0$ and the inequality in (A9) follows from $M_{L}\cdot \vec{u}\neq \vec{0}$ because $\vec{u}\neq \vec{0}$, and $M_{L}$ has full column rank. Thus, the inequality in (A5) is proven.

Next, we prove the “if” part. According to the security condition (4), we prove that

(A10) $$H\left(B\;\cdot \;M_{L}|Y_{W}\right)=H\left(B\;\cdot \;M_{L}\right),\;\;\forall \;W\in W_{r}$$

if the condition in (35) is satisfied. To prove (A10), it suffices to show that for each $W\in W_{r}$, the equality

(A11) $$\mathrm{Pr}\left(B\;\cdot \;M_{L}=\vec{x}|Y_{W}=\vec{y}\right)=\mathrm{Pr}\left(B\;\cdot \;M_{L}=\vec{x}\right)$$

is satisfied for any $\vec{x}\in F^{\tau L}$ row vector and any $\vec{y}\in F^{nr}$ row vector such that $\mathrm{Pr}\left(Y_{W}=\vec{y}\right)>0$, i.e., there exists a pair (bk) of a vectors of source symbols $b\in F^{L}$ and a key $k\in F^{z}$ such that $\left(b\;k\right)\cdot H_{W}=\vec{y}$.

We recall that

$$\begin{array}{c} \mathrm{Pr}\left(B\;\cdot \;M_{L}=\vec{x}\right)=\frac{1}{{\left|F\right|}^{\tau L}},\;\;\forall \;\vec{x}\in F^{\tau L} \end{array}$$

(cf. (10)). Thus, we only need to prove that for each $W\in W_{r}$,

$$\mathrm{Pr}\left(B\;\cdot \;M_{L}=\vec{x}|Y_{W}=\vec{y}\right)=\frac{1}{{\left|F\right|}^{\tau L}}$$

for any $\vec{x}\in F^{\tau L}\;\mathrm{and}\;\vec{y}\in F^{nr}\;\mathrm{such}\;\mathrm{that}\;\mathrm{Pr}\left(Y_{W}=\vec{y}\right)>0$. We now consider

(A12) $$\begin{array}{cc} \; & \mathrm{Pr}\left(B\;\cdot \;M_{L}=\vec{x}|Y_{W}=\vec{y}\right)\\ \; & =\frac{\mathrm{Pr}\left(B\;\cdot \;M_{L}=\vec{x},Y_{W}=\vec{y}\right)}{\mathrm{Pr}\left(Y_{W}=\vec{y}\right)}\\ \; & =\frac{\mathrm{Pr}\left(\left(B\;K\right)\;\cdot \;{\hat{M}}_{L}=\vec{x},\;\left(B\;K\right)\;\cdot \;H_{W}=\vec{y}\right)}{\mathrm{Pr}\left(\left(B\;K\right)\;\cdot \;H_{W}=\vec{y}\right)}\\ \; & =\frac{\mathrm{Pr}\left(\left(B\;K\right)\;\cdot \;\left[{\hat{M}}_{L}\;H_{W}\right]=\left(\vec{x}\;\;\vec{y}\right)\right)}{\mathrm{Pr}\left(\left(B\;K\right)\;\cdot \;H_{W}=\vec{y}\right)}\\ \; & =\frac{\sum_{\left(b\;k\right)\in F^{L}\times F^{z}:\;\left(b\;k\right)\left[{\hat{M}}_{L}\;H_{W}\right]=\left(\vec{x}\;\vec{y}\right)}\mathrm{Pr}\left(B=b,\;K=k\right)}{\sum_{\left(b^{\prime }\;k^{\prime }\right)\in F^{L}\times F^{z}:\;\left(b^{\prime }\;k^{\prime }\right)H_{W}=\vec{y}}\mathrm{Pr}\left(B=b^{\prime },\;K=k^{\prime }\right)}\\ \; & =\frac{\#\left\{\left(b\;k\right)\in F^{L}\times F^{z}:\left(b\;k\right)\;\cdot \;\left[{\hat{M}}_{L}\;H_{W}\right]=\left(\vec{x}\;\;\vec{y}\right)\right\}}{\#\left\{\left(b^{\prime }\;k^{\prime }\right)\in F^{L}\times F^{z}:\left(b^{\prime }\;k^{\prime }\right)\;\cdot \;H_{W}=\vec{y}\right\}}, \end{array}$$

where we use “#{·}” to denote the cardinality of the set, and the equality (A12) follows because B and K are independently and uniformly distributed on $F^{L}$ and $F^{z}$, respectively. Furthermore,

- For the denominator in (A12), we have
  (A13) $$\#\left\{\left(b^{\prime }\;k^{\prime }\right)\in F^{L}\times F^{z}:\left(b^{\prime }\;k^{\prime }\right)\;\cdot \;H_{W}=\vec{y}\right\}={\left|F\right|}^{L+z-\mathrm{Rank}(H_{W})};$$
- For the numerator in (A12), we have
  (A14) $$\begin{array}{cc} \; & \#\left\{\left(b\;k\right)\in F^{L}\times F^{z}:\left(b\;k\right)\;\cdot \;\left[{\hat{M}}_{L}\;H_{W}\right]=\left(\vec{x}\;\;\vec{y}\right)\right\}\\ \; & ={\left|F\right|}^{L+z-\mathrm{Rank}\left(\left[{\hat{M}}_{L}\;H_{W}\right]\right)}\\ \; & ={\left|F\right|}^{L+z-\mathrm{Rank}(H_{W})-\tau L}, \end{array}$$
  where the equality (A14) follows from the following condition: $\langle {\hat{M}}_{L}\rangle ⋂\langle H_{W}\rangle =\left\{\vec{0}\right\}$ (cf. (35)).

Combining (A13) and (A14) with (A12), we immediately prove that

$$\mathrm{Pr}\left(B\cdot M_{L}=\vec{x}|Y_{W}=\vec{y}\right)=\frac{1}{{\left|F\right|}^{\tau L}},$$

which implies the equality in (A11). The “if” part is also proven. We have thus proven the lemma.
